# Advances in detection methods for Shiga toxin-producing *Escherichia coli* (STEC)

**DOI:** 10.3389/fmicb.2014.00277

**Published:** 2014-06-05

**Authors:** Nora L. Padola

**Affiliations:** Inmunoquímica y Biotecnología-CIVETAN-CONICET-CIC-FCV-Universidad Nacional del Centro de la Provincia de Buenos AiresTandil, Argentina

**Keywords:** Shiga toxin-producing *E. coli*, diagnosis, serotypes, virulence genes, RT-PCR

Shiga toxin-producing *Escherichia coli* (STEC) comprise *E. coli* strains capable of producing toxins named Shiga toxin type 1 (Stx1), type 2 (Stx2), or both, encoded by *stx*1 and *stx*2 genes, respectively. Stx2 is the most heterogeneous group possessing subtypes that differ in their association with human disease (Friedrich et al., 2002). Other typical virulence factors are intimin (encoded by *eae* gene), a plasmid-encoded enterohemolysin and, in strains lacking *eae*, an autoagglutinating adhesin (Saa) (Paton and Paton, 2002; Blanco et al., 2004).

STEC are foodborne pathogens that have been associated worldwide with outbreaks and sporadic cases of hemolytic uremic syndrome (HUS) in children, STEC O157:H7 being the most notorious agent of the group. However, there are more than 400 non-O157 serotypes that have been involved in human disease and isolated from reservoirs of infection (Bettelheim, 2007; Rivas et al., 2010; Mora et al., 2011). It is important to note that not all non-O157 STEC have the capacity to cause HUS, which presents a public health problem to identify high-risk STEC.

STEC O157 and non-O157 have been isolated from meat, milk and dairy products, water, unpasteurized apple drinks, and vegetables. Additionally, direct contact with cattle and animal environment are currently considered as a source of transmission (Etcheverría and Padola, 2013).

Since 1982 when STEC O157:H7 was first reported, the selective diagnostic methods have used particular features of this serotype to increase its isolation. Because of the use of these selective methods, the prevalence of non-O157 STEC has been underestimated during several years. Both STEC O157 and non-O157 STEC have also been detected also using molecular methods that do not exert selection pressure toward any particular serotype, such as detecting *stx* in samples by PCR followed by isolation of colonies, detection of *stx1*, *stx2*, *eae*, *saa*, *ehxA* by multiplex PCR and serotyping (Scheutz et al., 2001; Fernández et al., 2010; Piazza et al., 2010) but this procedure is laborious and time consuming. PCR protocols have also been developed to detect particular serogroups and H antigens.

During 2012, Food Safety and Inspection Service (FSIS) determined the zero-tolerance policy for STEC O157:H7 including the top 6 non-O157 STEC serogroups in raw, nonintact beef products (Wang et al., 2013). Because of this, commercial test kits for screening from enrichment cultures based on immunological and molecular methods have been developed to detect *stx*, *eae*, and serogroups frequently associated with severe human disease (O157, O26, O103, O111, and O145). These enrichment samples could have individual bacteria or mixture of different bacteria harboring *stx*, *eae*, and serogroups that should be confirmed with isolation.

A large outbreak that ocurred in 2011 in Germany was caused by a strain O104:H4 harboring *stx2*. However, this strain was not a typical STEC because also carried a genome of enteroaggregative *E. coli* (EAEC) (Mellmann et al., 2011). *stx* genes are carried by lysogenic phages and could transfer to other bacteria by horizontal transfer. For this reason, STEC non-O157 are considered emerging pathogens that rapidly evolve and new serogroups of significant public health risk should be considered by diagnostic methods (Coombes et al., 2011). Indeed, the pathogenicity of STEC should not be restricted to a panel of serogroups or a single gene such as *eae* besides *stx* (EFSA Panel on Biological Hazards (BIOHAZ), 2013).

In order to evaluate a new PCR-real time technology based on simultaneous detection of multiple targets, Fratamico and Bagi (2012) have used a GeneDisc system. The GeneDisc consists of a disposable plastic disc of the size a compact disc with 36 microchambers preloaded with desiccated PCR primers and fluorescence labeled gene probes that use a GeneDisc cycler (Beutin et al., 2009). Fratamico and Bagi (2012) evaluated this technology to detect *stx1*, *stx2*, *eae*, *ehxA*, and O157 followed by a second GeneDisc assay targeting eight STEC serogroup: O26, O45, O91, O103, O111, O113, O121, O145 in ground beef. Notably, these authors have incorporated two important STEC serogroups: O91 and O113 that lack the *eae* gene but carry *ehxA*. Both serogroups have been related to HUS cases.

In this study two enrichment media were compared, buffered peptone water (BPW) recommended by GeneDisc and mTSB for STEC detection in the ground beef artificially inoculated. With both media, PCR results were similar and target genes were correctly identified with similar Ct values. Immunomagnetic separation (IMS) for serogroups O26, O45, O103, O111, O121, O145, O157, and plating onto Rainbow agar O157 were used and isolates were confirmed as the correct STEC serogroup. Serogroups O91 and O113 were not subjected to IMS and were more difficult to isolate on Rainbow Agar O157. All isolates were confirmed by conventional PCR targeting *stx*1, *stx*2, and *eae*. These authors have described the use of Rainbow Agar O157 for isolation of non-O157 because non-O157 strains produce colonies of different colors according to serogroup.

Fratamico and Bagi (2012) demonstrated that this technology is rapid, simple to use and suitable for screening ground beef and other foods, and could be adapted to detect other serogroups of high risk in public health.

## Conflict of interest statement

The author declares that the research was conducted in the absence of any commercial or financial relationships that could be construed as a potential conflict of interest.
